# Diagnostic, Prognostic, and Immunomodulatory Roles of Prostaglandin E Receptor 1 (PTGER1): A Pan-Cancer Study

**DOI:** 10.7759/cureus.99926

**Published:** 2025-12-23

**Authors:** Rasha Suliman, Mohamed Alfaki

**Affiliations:** 1 Medicine, University of Medical Sciences and Technology, Khartoum, SDN; 2 Software Engineering, Al Neelain University, Khartoum, SDN

**Keywords:** bioinformatics, cancer genetics, diagnostic biomarker, epigenetics, prognostic biomarker, ptger1

## Abstract

Background: The prostaglandin E receptor 1 (PTGER1) is a G-protein-coupled receptor (GPCR) located on chromosome 9. It encodes prostaglandin E2 receptor 1 (EP1), a 42kDa prostaglandin receptor involved in mediating many physiological and pathological responses to prostaglandin E2 (PGE2), including inflammation, pain perception, and modulation of cell proliferation. We performed a pan-cancer, multi-omics analysis of PTGER1 to evaluate its diagnostic, prognostic, and immunological relevance across human cancers.

Methodology: We used multiple public cancer bioinformatics platforms including the Tumor Immune Estimation Resource (TIMER), Gene Expression Profiling Interactive Analysis (GEPIA), and the University of ALabama at Birmingham CANcer Data Analysis Portal (UALCAN), Kaplan-Meier (KM-Plotter), CbioPortal and Gene Expression Omnibus (GEO) to evaluate PTGER1 expression, methylation, immune infiltration, genetic alteration and survival across tumor types.

Results: PTGER1 revealed significant (P < 0.05) expression in four cancer types; it was downregulated in kidney chromophobe (KICH) and kidney renal cell carcinoma (KIRC), uterine corpus endometrial cancer (UCEC), and upregulated in liver hepatocellular carcinoma (LIHC). Moreover, a significant hypermethylation of normal cells was noted in bladder carcinoma (BLCA), KIRC, and lung adenocarcinoma (LUAD). A significant hypermethylation of tumor cells in UCEC and kidney renal papillary (KIRP), and head and neck squamous carcinoma (HNSC) was noted. Interestingly, KICH showed staged methylation. PTGER1 demonstrated a statistically significant (P < 0.05), weakly positive correlation with immune cell populations, including CD8⁺ T cells, CD4⁺ T cells, dendritic cells, B cells, neutrophils, and macrophages in KIRC, KICH, and LIHC. Subsequently, prognostic analysis indicated that elevated PTGER1 expression was associated with poorer outcomes in KIRC and lung squamous cell carcinoma (LUSC) across three databases and improved prognosis in LIHC. From a genomic perspective, across a variety of cancers, PTGER1 was altered in approximately <2% (160 samples out of 10960 samples) primarily through gene amplification.

Conclusion: Analysis and cross-referencing across three publicly available databases revealed that PTGER1 is promising as a diagnostic biomarker in KICH and KIRC and a poor prognostic biomarker in KIRC and LUSC.

## Introduction

Cancer is a major global health challenge that affects millions of people each year. Despite advances in treatment, cancer continues to pose a significant threat to public health worldwide. Without urgent intervention, cancer diagnoses could rise to 30.5 million, and deaths may reach 18.6 million by 2050, with these nations facing the brunt of this crisis, accounting for over half of new cases and two-thirds of fatalities [1].

Pan-cancer analysis marks a pivotal shift in cancer research, as it seeks to unravel the complex genetic and molecular interconnections that exist among various cancer types. One critical component in this evolving landscape of cancer research is the analysis of molecular targets with unclear diagnostic and prognostic relevance across cancer types. In this context, the prostaglandin E receptor 1 (PTGER1) has emerged as a significant mediator of inflammation and cancer progression, yet its value as a diagnostic or prognostic biomarker has not been systematically investigated in the literature. PTGER1, a G protein-coupled receptor, mediates the effects of prostaglandin E2, a molecule that has been implicated in various malignancies by promoting tumor growth and metastasis through inflammatory pathways [2].

This study has two primary objectives: to evaluate PTGER1 as a diagnostic and prognostic biomarker across multiple cancer types, and to investigate its role in immune modulation and genetic variation. Utilizing publicly available datasets, including Gene Expression Profiling Interactive Analysis (GEPIA), the University of ALabama at Birmingham CANcer Data Analysis Portal (UALCAN), and the Tumor Immune Estimation Resource (TIMER), we systematically analyzed gene expression profiles, immune cell infiltration, survival outcomes, and prevalent genetic alterations. Our findings aim to enhance the understanding of PTGER1’s contribution to tumor pathogenesis, improve diagnostic and prognostic precision, guide the development of more effective and broadly applicable therapeutic strategies, and bridge the gap between basic research and clinical oncology [3-5].

## Materials and methods

PTGER1 gene expression profile across multiple malignancies

A comprehensive pan-cancer transcriptomic analysis of PTGER1 was conducted using an integrated multi-database approach. We initially employed TIMER (https://cistrome.shinyapps.io/timer/) for pan-cancer expression and immune infiltration analysis using The Cancer Genome Atlas (TCGA) data [3]. UALCAN data analysis portal (http://ualcan.path.uab.edu/), an interactive portal that integrates multi-omics cancer data, was used to analyse gene expression alterations across diverse malignancies, along with analysis of gene expression in relation to key clinicopathological features, including tumor stage, patient age, and demographics, utilizing level 3 RNA-sequencing data from TCGA [4]. GEPIA (http://gepia.cancer-pku.cn/) was employed to validate tumor-normal expression profiling by integrating malignancy-derived RNA-sequencing data from TCGA with healthy tissue controls from TCGA and GTEx databases [5].

Methylation of the promoter region of PTGER1

Promoter DNA methylation of PTGER1 was systematically analyzed using the UALCAN database (http://ualcan.path.uab.edu/), which integrates TCGA methylation data and enables robust comparative assessment of hypermethylation and hypomethylation patterns between tumor and normal tissues across diverse cancer types [4].

Tumor immunological contexture and correlation with PTGER1 expression

To characterize the immunological context of PTGER1 expression, we utilized TIMER (https://cistrome.shinyapps.io/timer/) to analyze associations between PTGER1 transcript abundance and the composition of tumor-infiltrating immune cells through transcriptome-based deconvolution of TCGA specimens. This computational framework generates abundance estimates for six principal immune cell categories, including B cells, CD4+ and CD8+ T lymphocyte compartments, macrophages, neutrophils, and dendritic cells [3].

PTGER1 as a prognostic factor for overall survival (OS)

The UALCAN database was incorporated to analyze PTGER1 expression and OS across various malignancies [4]. Subsequently, GEPIA was utilized to perform survival analyses, including OS and disease-free survival, and assess expression correlations with other genes of interest [5]. Lastly, the prognostic value of PTGER1 was investigated across multiple cancer types through Kaplan-Meier Plotter (https://kmplot.com/), which enables OS analysis based on gene expression data from large-scale tumor cohorts. We incorporated hazard ratios and adjusted P-values.

Integrative analysis of large-scale oncogenomic data

cBioPortal (http://cbioportal.org), an open-access web platform for cancer genomics, was employed to analyze and visualize genetic alterations, co-expression patterns, and clinical correlations across TCGA cancer cohorts. To assess the genomic landscape of PTGER1, we performed comprehensive mutation analysis across various cancer types using the cBioPortal platform [6].

Revalidation

The Gene Expression Omnibus (GEO) (https://www.ncbi.nlm.nih.gov/geo/geo2r) repository serves as an open-access database curated by the National Centre for Biotechnology Information (NCBI) (https://doi.org/10.1093/nar/gkab1112) for depositing and retrieving diverse functional genomics data, including transcriptomic profiles from microarray and next-generation sequencing platforms [7]. We employed GEO2R (NCBI, Bethesda, MD, USA), an online analytical tool that utilizes established R computational frameworks to compare two or more groups of samples to identify differentially expressed genes (DEGs) and generate significance-ranked expression profiles [8].

## Results

PTGER1 gene expression across multiple malignancies

**Figure 1:**
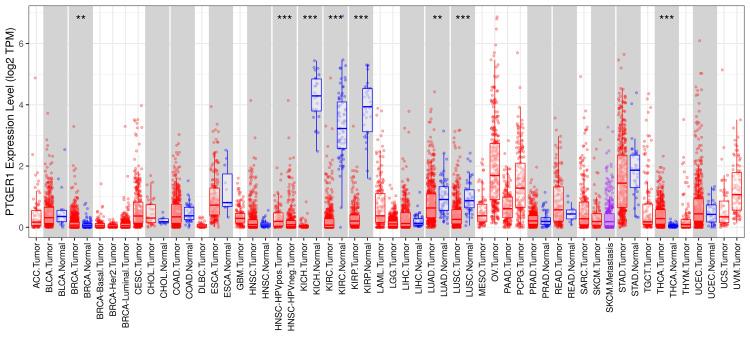
The figure presents a comparative overview of PTGER1 expression across a range of malignancies using TIMER1. Box plots display PTGER1 expression across tumor and normal tissues based on TCGA data. The x-axis represents expression levels (log_2_ TPM), while the y-axis lists tissue types. Each plot depicts the median, interquartile range, and outliers, with group differences evaluated using the Wilcoxon rank-sum test. *p < 0.05, **p < 0.01, ***p < 0.001, ****p < 0.0001; Red box: tumor; blue box: normal. TPM: transcripts per million; ACC: adrenocortical carcinoma, BLCA: bladder urothelial carcinoma, BRCA: breast invasive carcinoma, Her2: human epidermal growth factor 2, CESC: cervical squamous cell carcinoma and endocervical adenocarcinoma, CHOL: cholangiocarcinoma, COAD: colon adenocarcinoma, DLBC: lymphoid neoplasm diffuse large B-cell lymphoma, ESCA: esophageal carcinoma, GBM: glioblastoma multiforme, HNSC: head and neck squamous cell carcinoma, HPVpos: human papillomavirus positive, HPVneg: human papillomavirus negative, KICH: kidney chromophobe, KIRC: kidney renal clear cell carcinoma, KIRP: kidney renal papillary cell carcinoma, LAML: acute myeloid leukemia, LGG: brain lower grade glioma, LIHC: liver hepatocellular carcinoma, LUAD: lung adenocarcinoma, LUSC: lung squamous cell carcinoma, MESO: mesothelioma, OV: ovarian serous cystadenocarcinoma, PAAD: pancreatic adenocarcinoma, PCPG: pheochromocytoma and paraganglioma, PRAD: prostate adenocarcinoma, READ: rectum adenocarcinoma, SARC: sarcoma, SKCM: skin cutaneous melanoma, STAD: stomach adenocarcinoma, TGCT: testicular germ cell tumors, THCA: thyroid carcinoma, THYM: thymoma, UCEC: uterine corpus endometrial carcinoma, UCS: uterine carcinosarcoma, UVM: uveal melanoma, TIMER 1: Tumor Immune Estimation Resource, PTGER1: prostaglandin E receptor 1

PTGER1 expression was systematically profiled in tumor versus normal tissues using three publicly accessible platforms: TIMER, GEPIA, and UALCAN. Initial analysis using the TIMER database allowed a broad comparative assessment of PTGER1 expression across multiple human cancer types, providing foundational insights into its tumor-specific regulation. Comprehensive evaluation of PTGER1 expression across nine TIMER revealed distinct tumor-specific alterations. Notably, expression levels were significantly elevated in glioblastoma multiforme (GBM), head and neck squamous cell carcinoma (HNSC), liver hepatocellular carcinoma (LIHC), and uterine corpus endometrial carcinoma (UCEC) relative to corresponding normal tissues. Conversely, marked downregulation was observed in bladder urothelial carcinoma (BLCA), kidney chromophobe (KICH), kidney renal clear cell carcinoma (KIRC), lung adenocarcinoma (LUAD), and stomach adenocarcinoma (STAD) (Figure 1).

**Figure 2:**
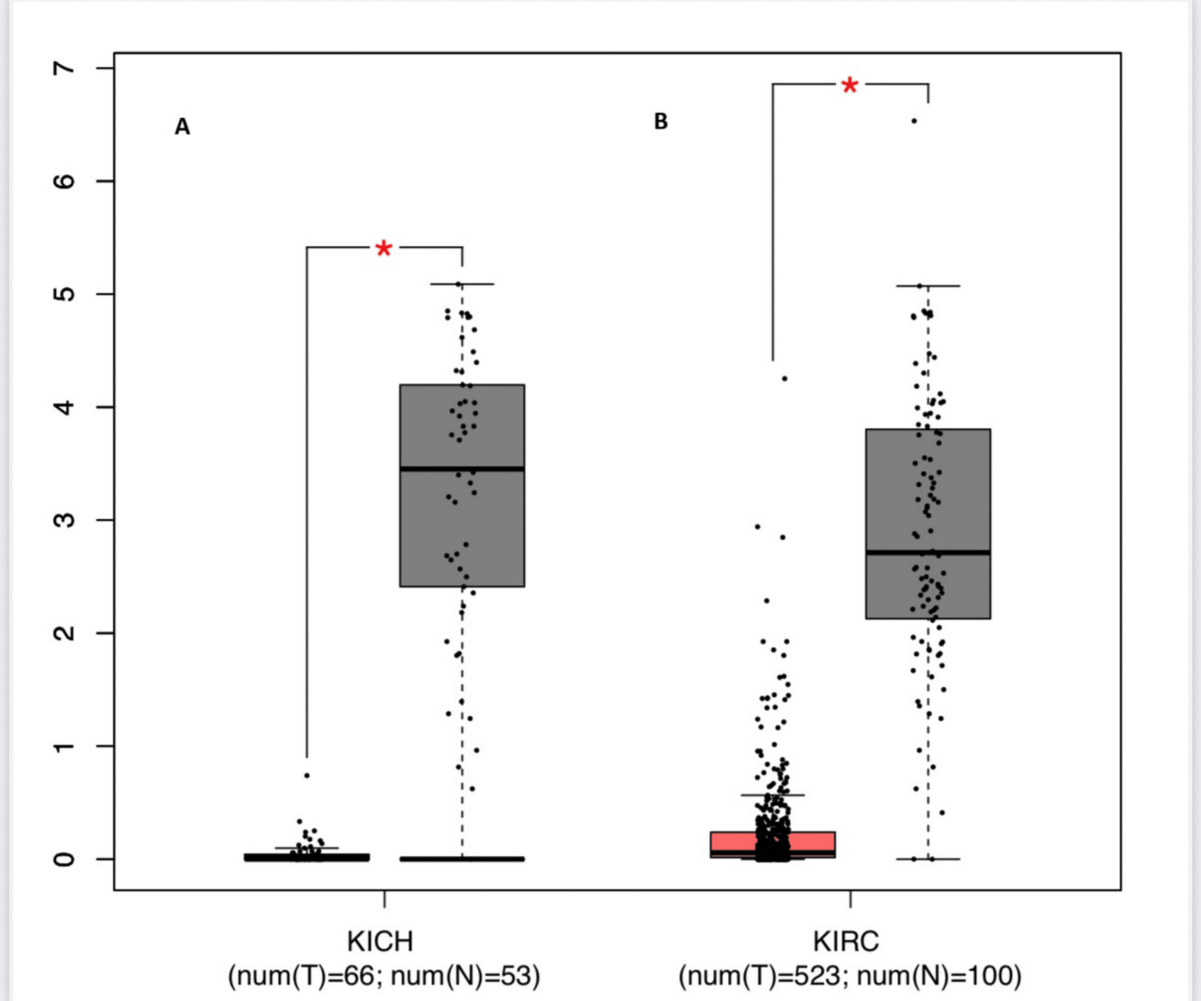
PTGER1 expression across various cancer types visualized using GEPIA. The figure compares tumor and normal tissue expression levels, highlighting significant dysregulation in selected malignancies. PTGER1 expression in (A) KICH, (B) KIRC. Y-axis: PTGER 1 expression level (log2 TPM). *p < 0.05, **p < 0.01, ***p < 0.001, ****p < 0.0001; Red: tumor, Blue: normal. KIRC: kidney renal cell carcinoma, KICH: kidney chromophobe, GEPIA: Gene Expression Profiling Interactive Analysis, PTGER1: prostaglandin E receptor 1

To further interrogate these findings, PTGER1 expression was assessed across the same set of tumors: GBM, LIHC, UCEC, BLCA, LUAD, STAD, KICH, KIRC, and HNSC, using the GEPIA platform. The analysis employed strict thresholds of (|log₂FC| ≥ 1.5; P < 0.05), where PTGER1 demonstrated statistically significant downregulation specifically in KICH and KIRC, reinforcing observations from complementary databases (Figure 2). In contrast, an insignificant downregulation of PTGER1 in tumor samples was observed in LUAD, BLCA, and GBM, and an insignificant upregulation in LIHC and UCEC.

**Figure 3:**
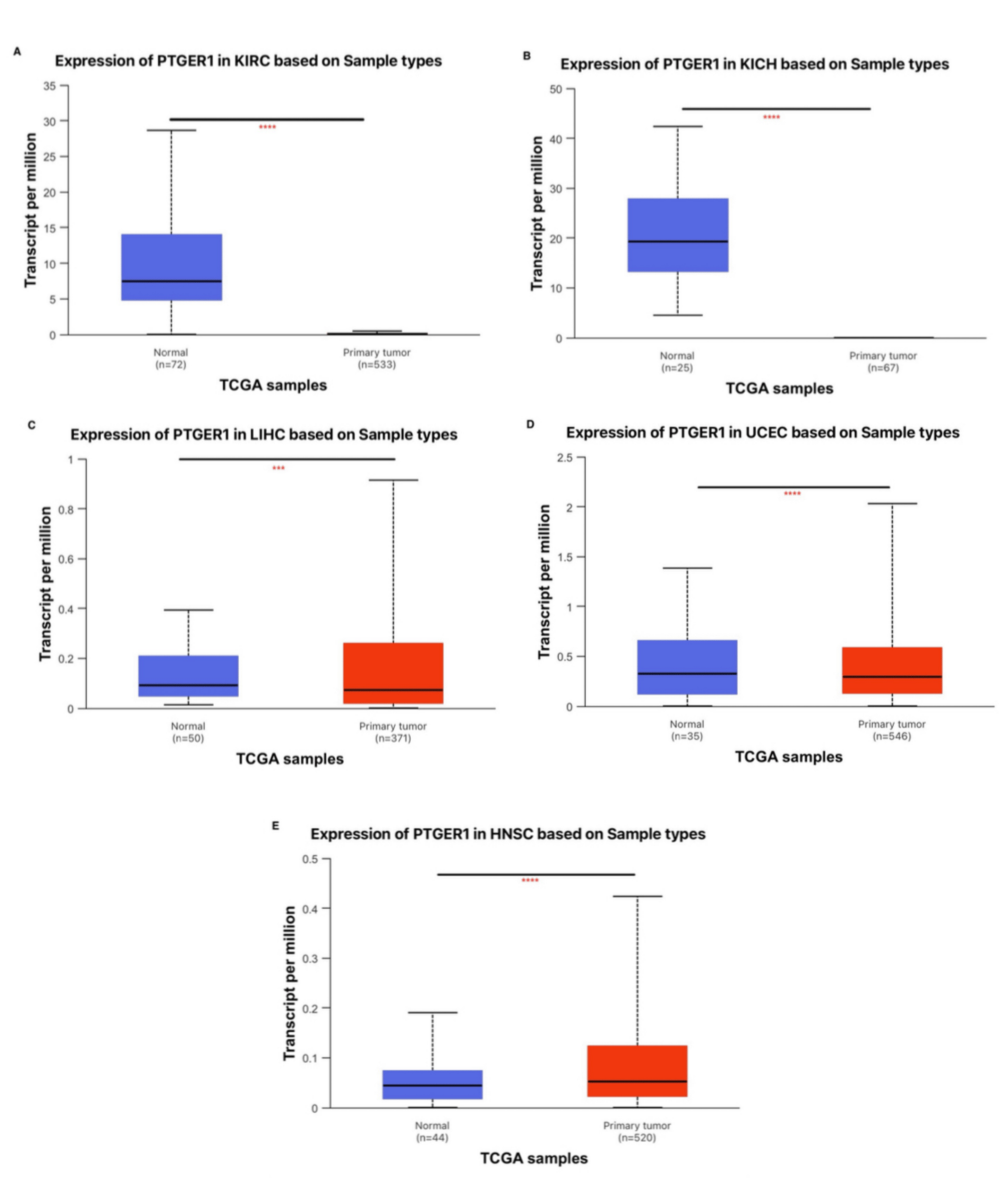
Differential expression patterns of PTGER1 in normal versus cancerous tissues across five cancer types, based on UALCAN analysis. PTGER1 expression in (A) KIRC, (B) KICH, (C) LIHC, (D) UCEC, and (E) HNSC. *p < 0.05, **p < 0.01, ***P<0.001, ****p < 0.0001; Red: tumor, Blue: normal. KIRC: kidney renal cell carcinoma, KICH: kidney chromophobe, LIHC: liver hepatocellular carcinoma, UCEC: uterine corpus endometrial carcinoma, HNSC: head and neck squamous carcinoma; UALCAN: University of ALabama at Birmingham CANcer Data Analysis Portal, PTGER1: prostaglandin E receptor 1, TCGA: The Cancer Genome Atlas

Analysis via UALCAN, applied as a final validation layer, highlighted cancer-specific modulation of PTGER1 expression across nine TIMER-identified malignancies. Notably, diminished expression was detected in renal cancers such as KICH (P<0.0001) and KIRC (P<0.0001), while statistically significant elevated levels were prominent in HNSC (p<0.0001), LIHC, and UCEC (p<0.0001), underscoring potential tumor type-specific regulatory mechanisms (Figure 3).

Overall, PTGER1 expression profiling across TIMER, GEPIA, and UALCAN consistently revealed significant downregulation in renal cancers, particularly KICH and KIRC, highlighting a reproducible suppression pattern across platforms. Upregulation in LIHC and UCEC was also supported by both TIMER and UALCAN, while in GEPIA, they were insignificant under strict statistical thresholds. These cross-platform findings suggest that PTGER1 expression may be cancer type specific, with renal, hepatic, and endometrial tumors showing the most consistent regulatory trends. PTGER1 emerges as a promising candidate diagnostic biomarker for renal cancers, due to its consistent and significant downregulation across three independent datasets: TIMER, GEPIA, and UALCAN. This suppression pattern suggests a potential role for PTGER1 in kidney-specific tumorigenesis or tumor suppression. Its differential expression between tumor and normal tissues, observed across distinct analytical platforms, supports its diagnostic utility and highlights its relevance in renal malignancies. Further validation through cross-platform bioinformatics analysis and clinical correlation could strengthen its applicability in cancer detection strategies.

Association between PTGER1 expression and clinicopathological features in cancer

**Figure 4:**
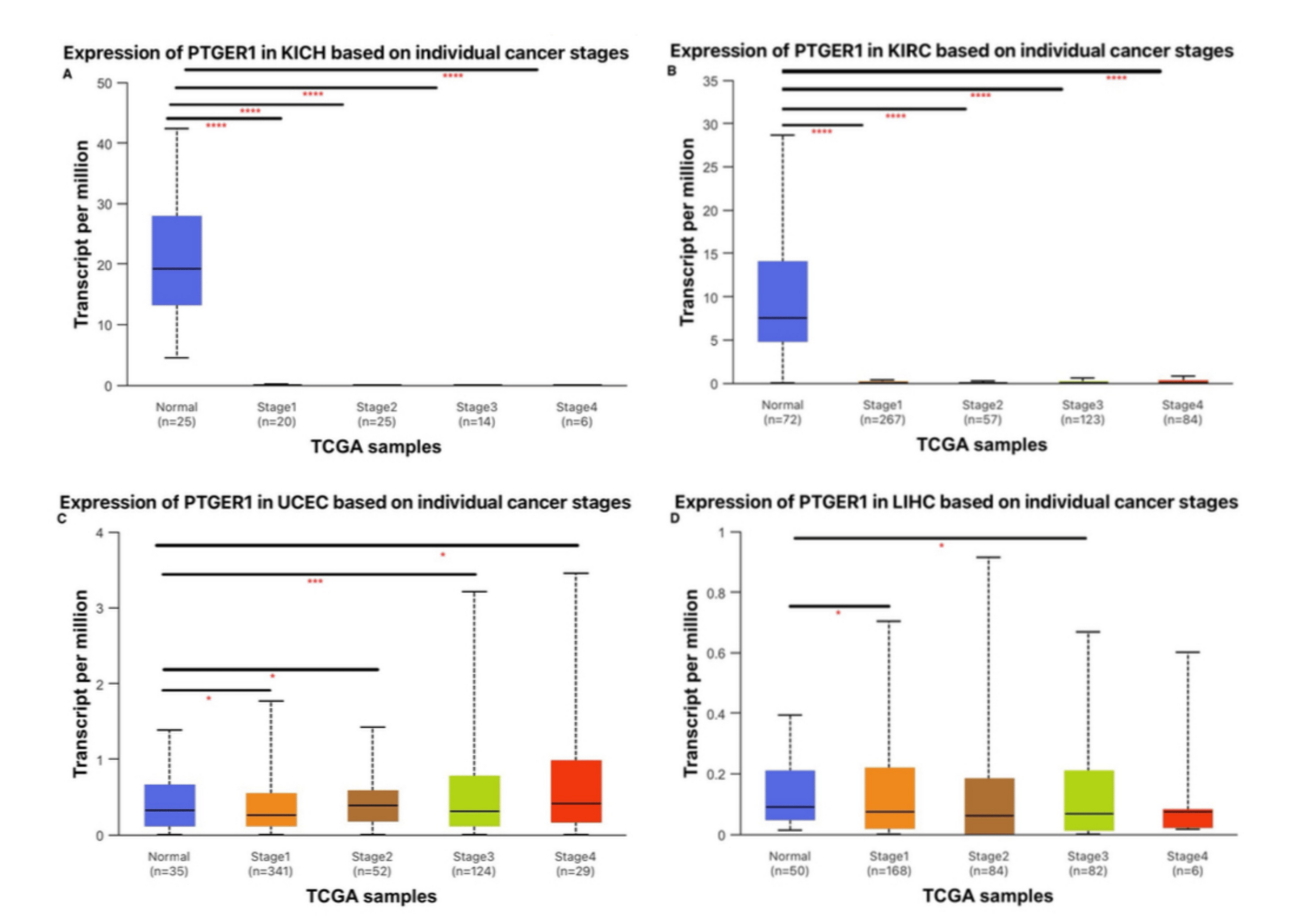
Stage-specific analysis of PTGER1 expression via UALCAN. *p < 0.05, **p < 0.01, ***P<0.001, ****p < 0.0001. PTGER1 expression in (A) KICH, (B) KIRC, (C) UCEC, and (D) LIHC. KIRC: kidney renal cell carcinoma, KICH: kidney chromophobe, UCEC: uterine corpus endometrial carcinoma, LIHC: liver hepatocellular carcinoma, PTGER1: prostaglandin E receptor 1, UALCAN: University of ALabama at Birmingham CANcer Data Analysis Portal, TCGA: The Cancer Genome Atlas

PTGER1 expression was further interrogated using the UALCAN database, focusing on cancer types that exhibited concordant expression patterns across at least two independent platforms utilizing GEPIA, UALCAN, and TIMER. In-depth classification-based analysis was conducted for KIRC, KICH, LIHC, and UCEC, where PTGER1 expression was systematically assessed in relation to key clinicopathological variables, including patient age, race, and tumor stage, providing insight into its potential demographic and disease-specific regulatory roles. PTGER1 expression was examined in tumor samples from patients spanning the third to 10th decades of life against normal tissues, categorized into four age cohorts: (21-40 years) young adults, (41-60 years) middle-aged, (61-80 years) older adults, and (81-100 years) elderly; across racial groups (Caucasian, African American, and Asian) vs. normal; and by tumor stage (1-4), which included normal versus each stage of tumor comparisons, as well as pairwise evaluations among different pathological stages. In terms of tumor staging, PTGER1 expression showed a significant difference between normal and tumor tissues in both KICH and KIRC (P<0.0001), with elevated expression levels in normal tissues and dramatic downregulation in tumor samples. This suggests a tumor suppressive role which may confidently be used as a diagnostic marker for renal cancer, particularly for distinguishing normal from malignant tissue. Further analysis revealed a stage-specific upregulation in KIRC, notably higher in stage 3 vs. stage 2 and in stage 4 vs. stage 2. In UCEC, stage-dependent patterns were noted with statistically significant upregulation of PTGER1 in normal tissues compared with stage 1 and stage 2, along with observed downregulation in normal tissues when compared with stages 3 and 4. Additionally, stage 3 showed a significant upregulation relative to stage 1, while stage 4 showed the highest trend. PTGER1 expression in UCEC increases consecutively across tumor stages, suggesting a potential role in late-stage tumor diagnosis, though this pattern warrants further validation. In contrast, LIHC displays a non-linear trend. PTGER1 expression peaks in stage 1, experiences a slight downregulation in stage 2, upregulates again in stage 3, and declines sharply by stage 4, where expression is minimal. A significant upregulation of PTGER1 in stage 1 and stage 3 was observed in LIHC when compared to normal samples. Notably, normal liver tissue exhibits moderate expression, slightly lower than in stage 1 tumors. This pattern suggests PTGER1 may be transiently upregulated during early and intermediate tumor stages, possibly reflecting a role in tumor initiation or inflammation and its potential use as a diagnostic biomarker for early-stage liver cancer, particularly stage 1 and stage 3. However, its declining expression in later stages limits its potential use as a marker for advanced LIHC (Figure 4).

**Figure 5:**
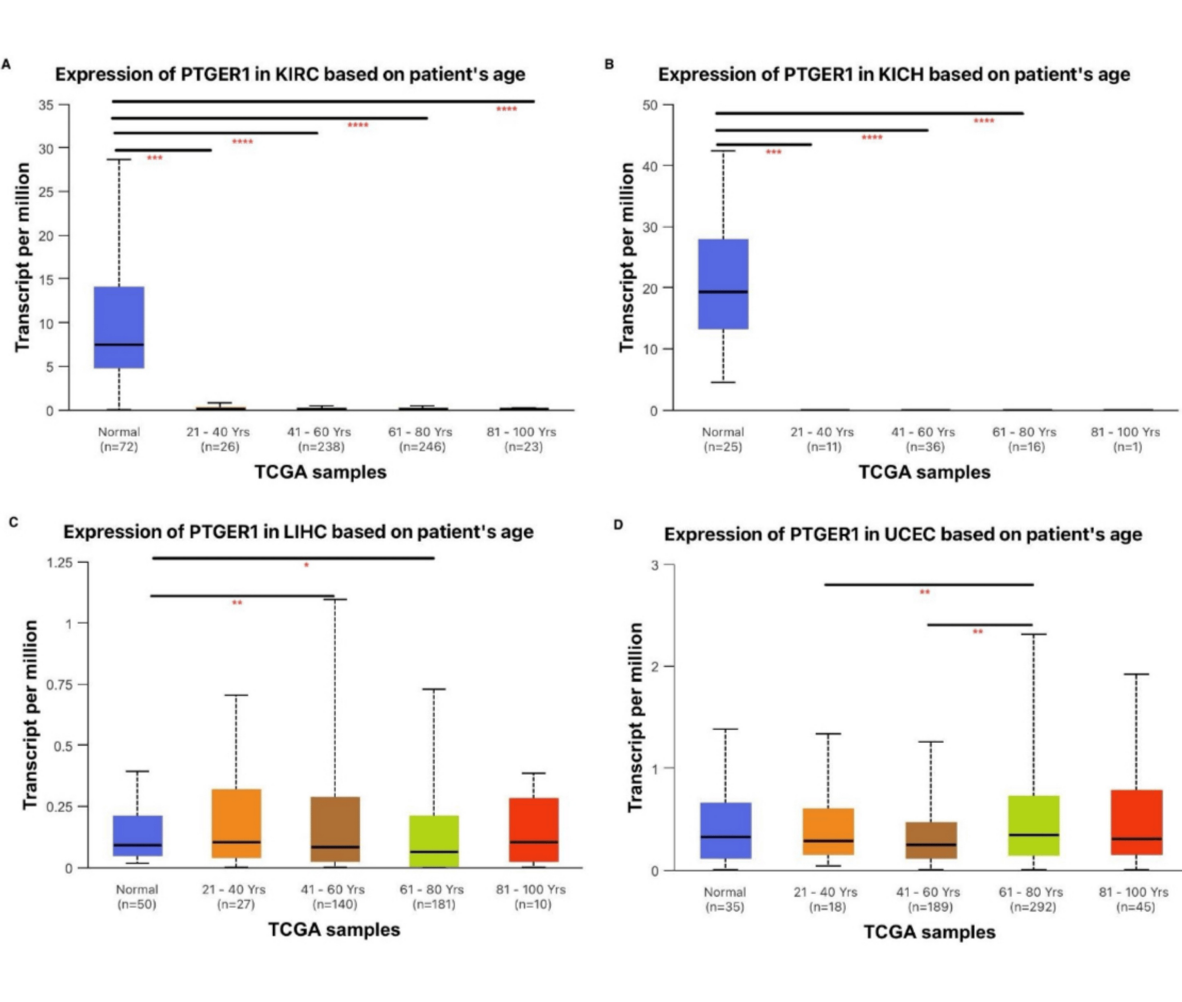
Age-specific analysis of PTGER1 expression via UALCAN. *p < 0.05, **p < 0.01, ***P<0.001, ****p < 0.0001. PTGER1 expression in (A) KIRC, (B) KICH, (C) LIHC, and (D) UCEC. KIRC: kidney renal cell carcinoma, KICH: kidney chromophobe, LIHC: liver hepatocellular carcinoma, UCEC: uterine corpus endometrial carcinoma, UALCAN: University of ALabama at Birmingham CANcer Data Analysis Portal, PTGER1: prostaglandin E receptor 1, TCGA: The Cancer Genome Atlas

Beyond tumor stage, patient age is a critical factor influencing gene expression profiles and the accuracy of diagnostic biomarkers. PTGER1 expression in KICH was significantly (P<0.0001) elevated in normal samples when compared with patients aged 21-40 years, 41-60 years, and 61-80 years, where expression was constantly downregulated. In KIRC, statistically significant (P<0.0001) differences were identified between normal tissue and tumor samples in patients aged 21-40 years, 41-60 years, 61-80 years, and 81-100 years, demonstrating a consistent downregulation of PTGER1 irrespective of age (Figure 5). This age-independent suppression supports the potential utility of PTGER1 as a diagnostic biomarker. PTGER1 expression in UCEC was significantly higher in elderly patients (61-80 years) compared to both younger (21-40 years), middle-aged (41-60 years) groups, and normal tissue. This indicates a notable upregulation of PTGER1 in the elderly UCEC population. The observed age-dependent increase in PTGER1 expression suggests that its regulation is influenced not only by tumor stage but also by aging, as its expression is not static across the lifespan. The age-associated increase in PTGER1 expression supports its potential as a diagnostic marker for age-related endometrial cancer. PTGER1 expression in LIHC shows mild age-associated upregulation, with statistically significant increases in middle-aged (41-60 years) and elderly patients (61-80 years) when compared to normal tissue. These may reflect biological changes associated with aging in liver tumors. The importance of patient age in clinicopathological analyses is essential to ensure that the evaluation of a gene’s potential as a diagnostic biomarker is accurate, reliable, and broadly applicable. This approach promotes diagnostic precision, safeguards patient safety, and supports consistent clinical decision-making across all age groups.

**Figure 6:**
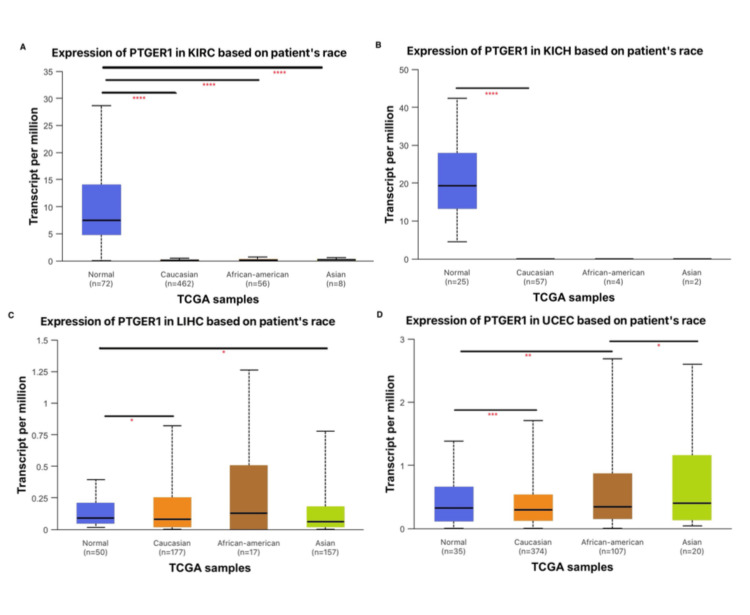
Race-specific analysis of PTGER1 expression via UALCAN database. *p < 0.05, **p < 0.01, ***P<0.001, ****p < 0.0001. PTGER1 expression in (A) KIRC, (B) KICH, (C) LIHC, and (D) UCEC. KIRC: kidney renal cell carcinoma, KICH: kidney chromophobe, LIHC: liver hepatocellular carcinoma, UCEC: uterine corpus endometrial carcinoma, UALCAN: University of ALabama at Birmingham CANcer Data Analysis Portal, PTGER1: prostaglandin E receptor 1, TCGA: The Cancer Genome Atlas

Racial and ethnic background is an important factor in diagnostic biomarker evaluation, as genetic diversity can influence expression patterns and disease traits. In KICH, PTGER1 expression was significantly (P<0.0001) upregulated in normal tissue compared to Caucasian tumor samples and showed an insignificant upregulation in normal samples when compared with African American patients and Asian patients. Similarly, in KIRC, a statistically significant difference was observed between normal tissue and tumor samples from Caucasian, African American, and Asian patients, with consistently higher expression in normal tissue (Figure 6). LIHC showed a statistically significant upregulation of PTGER1 in Caucasian patients when compared with normal samples, in addition to upregulation in normal samples when compared with the Asian population. The racial group with the highest expression and variability is African American patients, suggesting possible population-specific regulation of PTGER1 in LIHC. This racial variation points to the importance of including diverse populations in biomarker studies to ensure accurate interpretation and equitable diagnostic application across all patient groups. PTGER1 expression in UCEC showed a statistically significant difference across racial groups; normal tissue samples had higher expression compared to Caucasian tumor samples. In contrast, normal tissues showed a significantly lower expression when compared to African American patients, suggesting upregulation in tumor samples. Additionally, PTGER1 expression in UCEC was higher in Asian patients compared to African American patients. These findings indicate that PTGER1 expression in UCEC varies by race, leading to the importance of considering racial background when evaluating biomarkers to ensure diagnostic accuracy and equitable clinical outcomes across diverse populations. Utilizing the UALCAN platform to incorporate analyses of race, age, and tumour characteristics into clinicopathological studies offers a comprehensive approach to understanding gene expression patterns. This integration provides valuable insights into population-specific variations, enhances the reliability of biomarker evaluation, and supports the development of more inclusive, accurate, and personalized diagnostic strategies in cancer research and clinical practice (Figure 6).

Epigenetic regulation of PTGER1 and methylation patterns in human cancers

**Figure 7:**
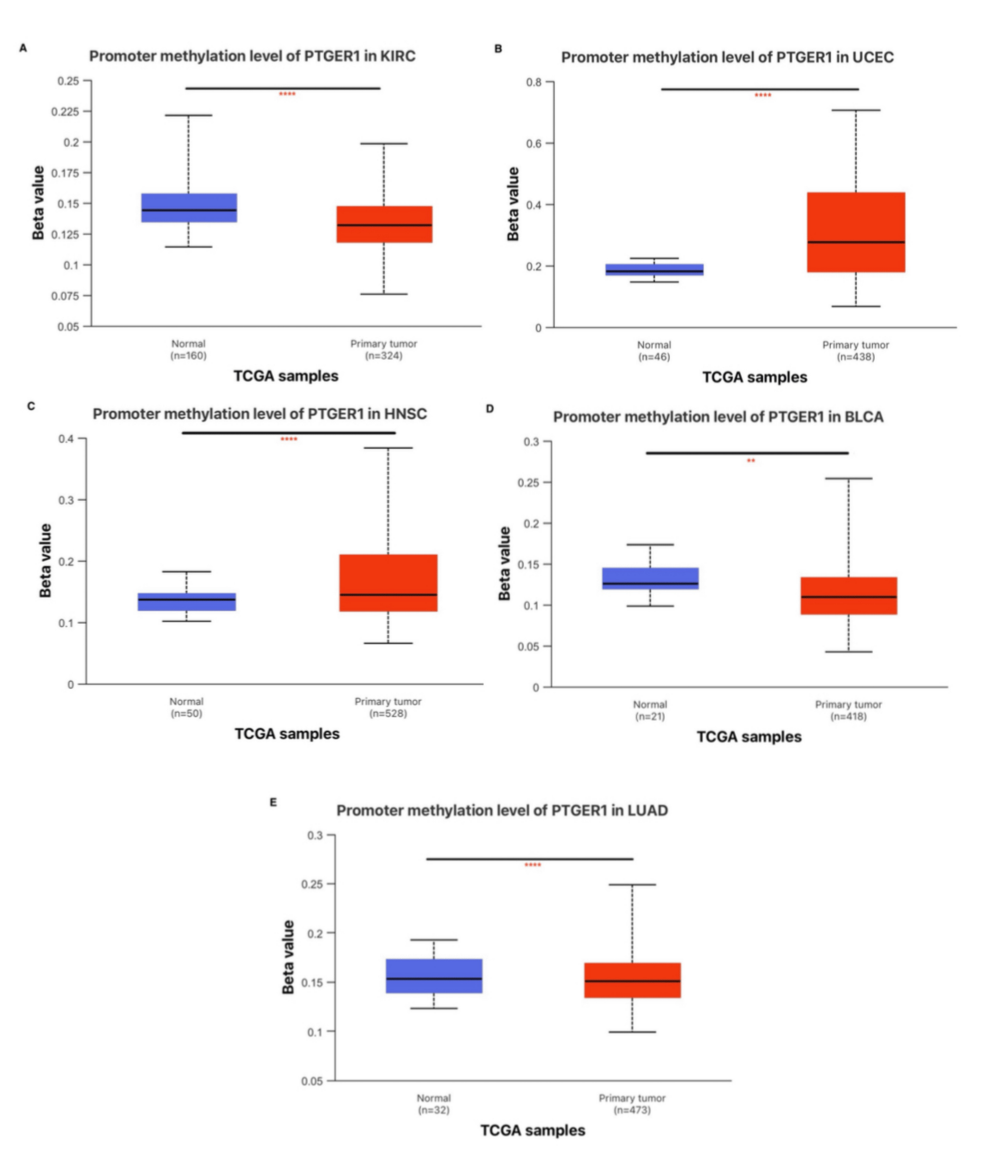
Comparative analysis of methylation status using UALCAN. *p < 0.05, **p < 0.01, ***P<0.001, ****p < 0.0001 PTGER1 methylation expression in (A) KIRC, (B) UCEC, (C) HNSC, (D) BLCA, and (E) LUAD. KIRC: kidney renal cell carcinoma, KICH: kidney chromophobe, LUAD: lung adenocarcinoma, UCEC: uterine corpus endometrial carcinoma, HNSC: head and neck squamous carcinoma, BLCA: bladder carcinoma, UALCAN: University of ALabama at Birmingham CANcer Data Analysis Portal, PTGER1: prostaglandin E receptor 1, TCGA: The Cancer Genome Atlas

Analysis of methylation in the promoter region of PTGER1 across multiple cancer types revealed distinct epigenetic regulation profiles. In BLCA, PTGER1 was hypermethylated in normal tissues, while tumor tissues were hypomethylated, correlating with its upregulation in tumors, suggesting that loss of methylation may drive increased expression. Conversely, in HNSC, despite being hypermethylated in tumors, PTGER1 expression was still elevated, indicating that methylation is not the primary regulatory mechanism in this context. In KIRC and LUAD, hypermethylation was observed in normal tissues alongside higher PTGER1 expression. In KICH, an insignificant PTGER methylation status in stage 1 revealed high variability, while stages 2 and 3 revealed intermediate levels; stage 4 exhibited elevated methylation compared to intermediate stages, suggesting potential changes in PTGER1 methylation across disease progression. Lastly, a statistically significant (P<0.0001) hypermethylation of PTGER1 occurred in UCEC tumor tissue samples, while higher expression in normal tissues aligns with hypomethylation-driven regulation, supporting methylation as a suppressive mechanism in this case (Figure 7).

Tumor immunological contexture and correlation with PTGER1 expression

**Figure 8:**
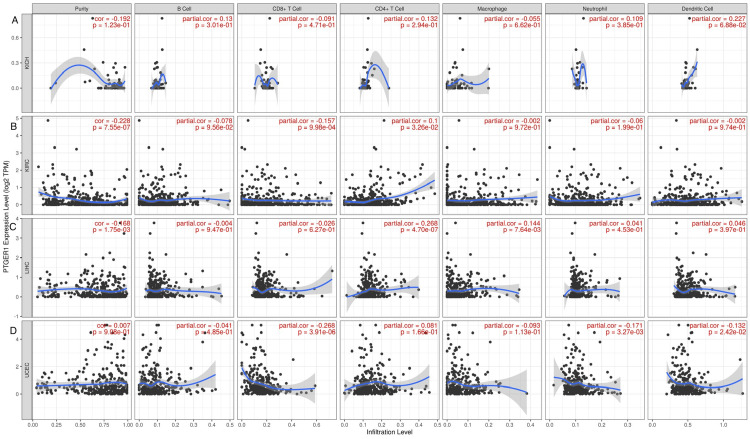
Correlation between PTGER1 expression and immune cell infiltration via TIMER. This figure shows PTGER1 expression correlation with immune cell infiltration, including CD8+ T cells, CD4+ T cells, macrophages, and dendritic cells. (A) KICH, (B) KIRC, (C) LIHC, and (D) UCEC. KIRC: kidney renal cell carcinoma, KICH: kidney chromophobe, LIHC: liver hepatocellular carcinoma, UCEC: uterine corpus endometrial cancer, TIMER: Tumor Immune Estimation Resource, PTGER1: prostaglandin E receptor 1

The relationship between PTGER1 expression and the immune microenvironment was evaluated across multiple human cancer types. Using the TIMER database, we analyzed the infiltration levels of key immune cell populations, including CD8+ cytotoxic T cells, CD4+ helper T cells, neutrophils, macrophages, and dendritic cells. Partial correlation analysis was employed to assess the strength and direction of associations between PTGER1 expression and immune cell infiltration. Correlation coefficients were categorized as strong (0.70-1.00), moderate (0.40-0.69), or weak (0.00-0.39), and further classified as positive (partial correlation > 0) or negative (partial correlation < 0). A positive correlation implies that increased PTGER1 expression is associated with higher immune cell infiltration, whereas a negative correlation suggests an inverse relationship. This approach provides a comprehensive view of PTGER1's potential role in modulating the tumor immune microenvironment. We conducted an analysis of four cancer types: KICH, KIRC, UCEC, and LIHC, which demonstrated statistical significance in at least two or more publicly available databases, including TIMER, GEPIA, and UALCAN. In KICH, PTGER1 expression showed a statistically significant (P < 0.05) weak positive correlation with CD4+ T cells (r: 0.099, P: 0.032) and a weak negative correlation with CD8+ T cells (r: -0.156, P: 0.0009). In addition, insignificant weak negative correlations were observed with B cells (r: -0.227, P: 0.095), macrophages (r: -0.0016, P: 0.97), neutrophils (r: -0.060, P: 0.198), and dendritic cells (r: -0.0015, P: 0.974), while KIRC, PTGER1 expression showed a statistically significant weak positive correlation with CD4+ T cells (r: 0.099, P: 0.0325). Following this, UCEC showed a statistically significant (P < 0.05) weak negative correlation of PTGER1 with neutrophils (r: -0.171, P: 0.0032) and dendritic cells (r: -0.131, P: 0.024). Statistically insignificant weak negative correlations were also found with B cells (r: -0.041, P: 0.485), CD8+ T cells (r: -0.267, P: 3.91133109344291e-06), and macrophages (r: -0.092, P: 0.113), alongside an insignificant weak positive correlation with CD4+ T cells (r: 0.081, P: 0.165). Lastly, in LIHC, immune infiltration analysis revealed a significantly weak positive correlation with macrophages (r: 0.144, P: 0.0076) (Figure 8).

The role of PTGER1 expression as a prognostic biomarker for patient survival outcome

**Figure 9:**
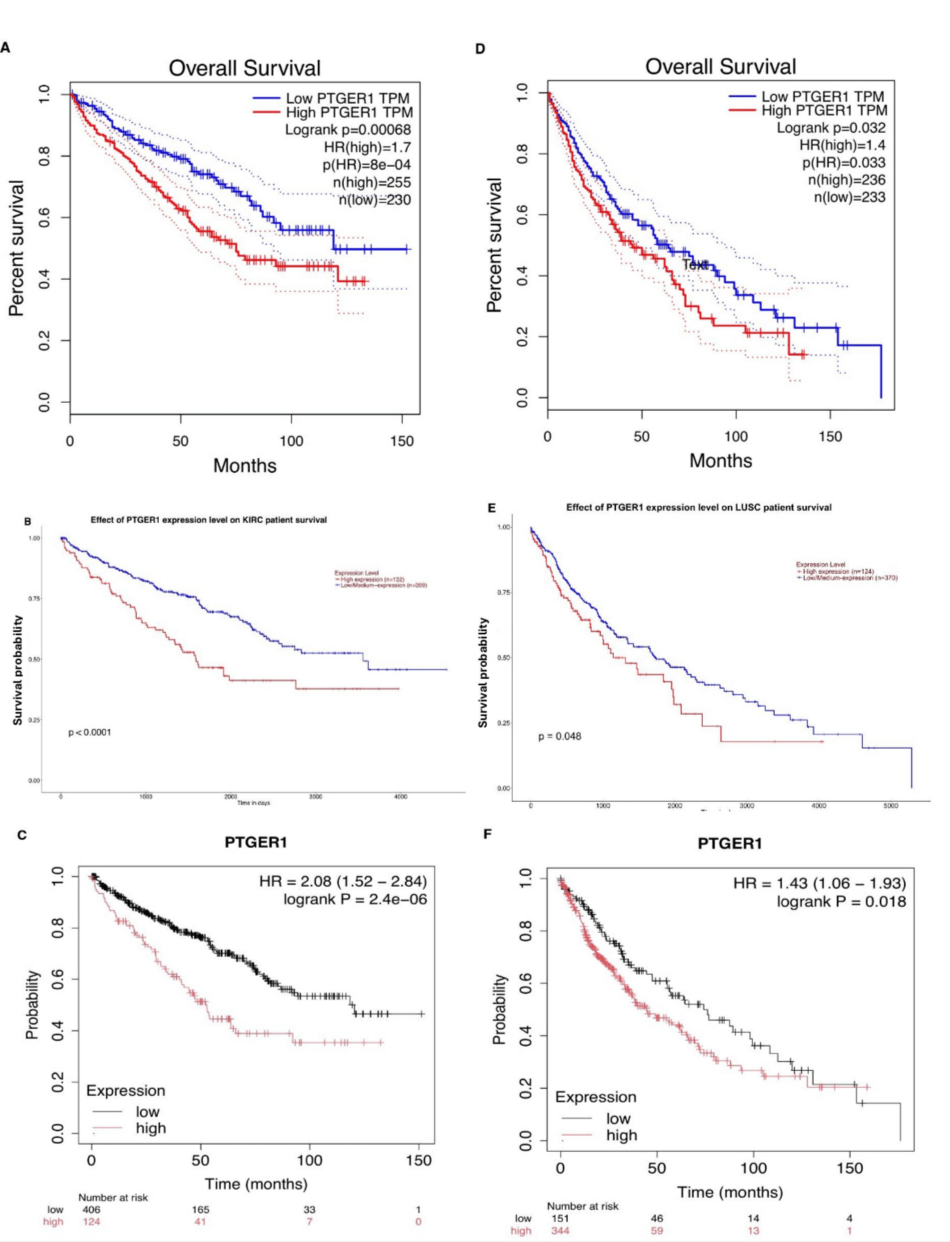
Prognostic impact of PTGER1 expression in KIRC and LUSC based on UALCAN, GEPIA and Kaplan Meier (KM) survival analysis. Comparative assessment of PTGER1 overall survival (OS) in KIRC using data from (A) GEPIA, (B) UALCAN, and (C) KM plots. Similar OS evaluation for PTGER1 in LUSC derived from (D) GEPIA 1, (E) UALCAN, and (F) Kaplan-Meier analysis. TPM: transcripts per million, Logrank: significance difference between low expression and high expression, n(high): total number of patients with high PTGER1 expression, n(low): total number of patients with low PTGER1 expression, HR-P-value: significance of hazard ratio, Red line: high expression, Blue line: low expression KIRC: kidney renal cell carcinoma, LUSC: lung squamous cell carcinoma, GEPIA: Gene Expression Profiling Interactive Analysis, UALCAN: University of ALabama at Birmingham CANcer Data Analysis Portal, PTGER1: prostaglandin E receptor 1

OS analysis of PTGER1 expression was performed using three publicly available databases: Kaplan-Meier Plotter (KM), GEPIA, and UALCAN. Utilizing the GEPIA database, OS was assessed with a median expression cut-off (50% high expression vs. 50% low expression). Hazard ratios (HRs) were calculated based on the Cox proportional hazards (Cox PH) model with a 95% confidence interval (CI), and survival time was measured in months. The results showed that KICH (Log-rank p: 0.0085, HR(high): 0.1, p(HR): 0.032) and LIHC (Log-rank p: 0.062, HR(high): 0.71, p(HR) = 0.064) were associated with a favourable prognosis in patients with high PTGER1 expression. In contrast, poor prognosis was observed in KIRC (Log-rank p: 0.00068, HR (high): 1.7, p(HR): 8e-04), esophageal cancer (ESCA) (Log-rank p: 0.05, HR: 0.63, p(HR: 0.051), low grade glioma (LGG) (Log-rank p: 0.028, HR (high): 1.5, p (HR): 0.029), lung squamous cell carcinoma (LUSC) (Log-rank p: 0.032, HR(high): 1.4, p (HR): 0.033), UVM (Log-rank p: 0.0081, HR(high): 3.4, p(HR): 0.012). Subsequently, UALCAN-based OS analysis revealed that elevated PTGER1 expression was significantly associated with poor survival in eight cancer types: head and neck squamous carcinoma (HNSC) (p = 0.032), KIRC (p < 0.0001), kidney renal papillary cell carcinoma (KIRP) (p = 0.043), LGG (p = 0.029), LUSC (p = 0.048), mesothelioma (MESO) (p = 0.00031), UCEC (p = 0.00045), and UVM (p < 0.0001). Further analysis using KM indicated that higher PTGER1 expression was associated with a favourable prognosis in five cancers: breast invasive carcinoma (BRCA) (Log-rank p = 0.054, HR = 0.8), ESCA (Log-rank p = 0.014, HR = 0.45), HNSC (Log-rank p = 0.07, HR = 0.78), LIHC (Log-rank p = 0.004, HR = 0.6), and thyroid carcinoma (THCA) (Log-rank p = 0.0014, HR = 0.22). Conversely, poor prognosis was observed in six cancer types: BLCA (Log-rank p = 0.078, HR = 0.77), KIRC (Log-rank p = 1.2e-08, HR = 2.35), KIRP (Log-rank p = 0.0038, HR = 2.35), LUSC (Log-rank p = 0.0044, HR = 1.59), Sarcoma (SARC) (Log-rank p = 0.0044, HR = 1.59), and UCEC (Log-rank p = 0.00049, HR = 2.06). Cross-referencing across two or more databases showed that high PTGER1 expression was consistently linked to poor prognosis in several cancers. KIRC and LUSC provided the strongest evidence for PTGER1 as a poor prognostic biomarker, with consistent findings confirmed across all three databases: GEPIA, UALCAN, and KM. UCEC, LGG, and UVM showed potential for PTGER1 to act as a poor prognostic biomarker; however, these associations were supported by two databases and therefore require further validation. In contrast, LIHC stands out, suggesting a possible role for PTGER1 as a good prognostic biomarker, although this finding was likewise limited to two databases and warrants additional confirmation (Figure 9).

Integrative analysis of large-scale oncogenomic data

**Figure 10:**
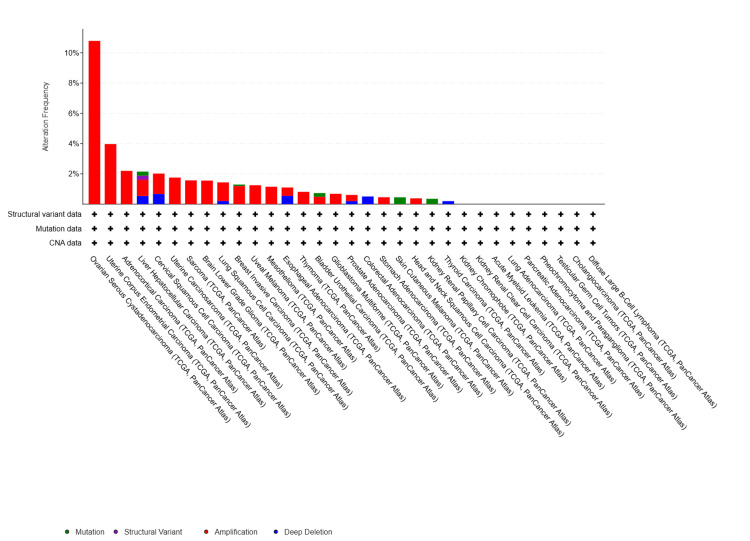
cBioPortal-based analysis of PTGER1 alteration frequencies across diverse cancer types. Data represent the proportion of samples within each cancer type harbouring mutations, amplifications, or deletions in PTGER1. PTGER1: prostaglandin E receptor 1

PTGER1 was analyzed using cBioPortal, an open-access, web-based platform that enables exploration of gene alterations across multiple cancer types to investigate its mutational landscape, expression patterns, and potential clinical relevance. This analysis integrates data from large-scale cancer genomics studies to uncover associations between PTGER1 aberrations and oncogenic processes. Across 32 cancer studies in the TCGA pan-cancer dataset, analysis of 10,967 samples from 10,953 patients revealed that PTGER1 harbours mutations in approximately 3% of cases. PTGER1 was altered in 160 (2%) of the queried samples. Many of these alterations were gene amplifications, followed by deep deletions, missense mutations of unknown significance, and a smaller number of structural variants. Samples were comprehensively profiled for copy number changes, mutations, and structural variations, ensuring a high level of confidence in the alteration landscape. The highest frequency of alteration was observed in ovarian serous cystadenocarcinoma (OV, over 10%) and UCEC (4%). Moderate alteration rates (2-3%) occurred in adrenocortical carcinoma (ACC), LIHC, and cervical squamous cell carcinoma (CESC), while most other cancers showed <2% frequency alteration. Alterations were primarily driven by amplification in eleven tumor types, with the highest rate in OV, where genetic alteration was detected in 10.79% (63/584) patients, followed by UCEC with 3.97% (21/529), ACC 2.2% (2/91), uterine carcinosarcoma (UCS) 1.75% (1/57), sarcoma (SARC) 1.57% (4/255), UVM 1.25% (1/80), MESO 1.15% (1/87), thymoma (THYM) 0.81% (1/123), GBM 0.68% (4/585), stomach adenocarcinoma (STAD) 0.45% (2/440), and head and HNSC 0.38% (2/523). In contrast, deep deletions contributed to a smaller fraction of cases, occurring solely in THCA 0.2% (1/499) and colorectal adenocarcinoma (COAD) 0.51% (3/594). Mutations were detected in two tumors: skin cutaneous melanoma (SKCM) 0.45% (2/442) and KIRP carcinoma 0.35% (1/283). A mix of amplification and deletion was observed in four tumor types: CESC, lung squamous cell carcinoma (LUSC), ESCA, and prostate adenocarcinoma (PRAD), with amplification predominating over deletion. Additionally, BLCA exhibited a mix of mutation and amplification, with amplification being the predominant alteration. Interestingly, in LIHC, PTGER1 exhibited a diverse alteration profile including amplification, deletion, structural variants, and mutations (Figure 10).

Revalidation

**Figure 11:**
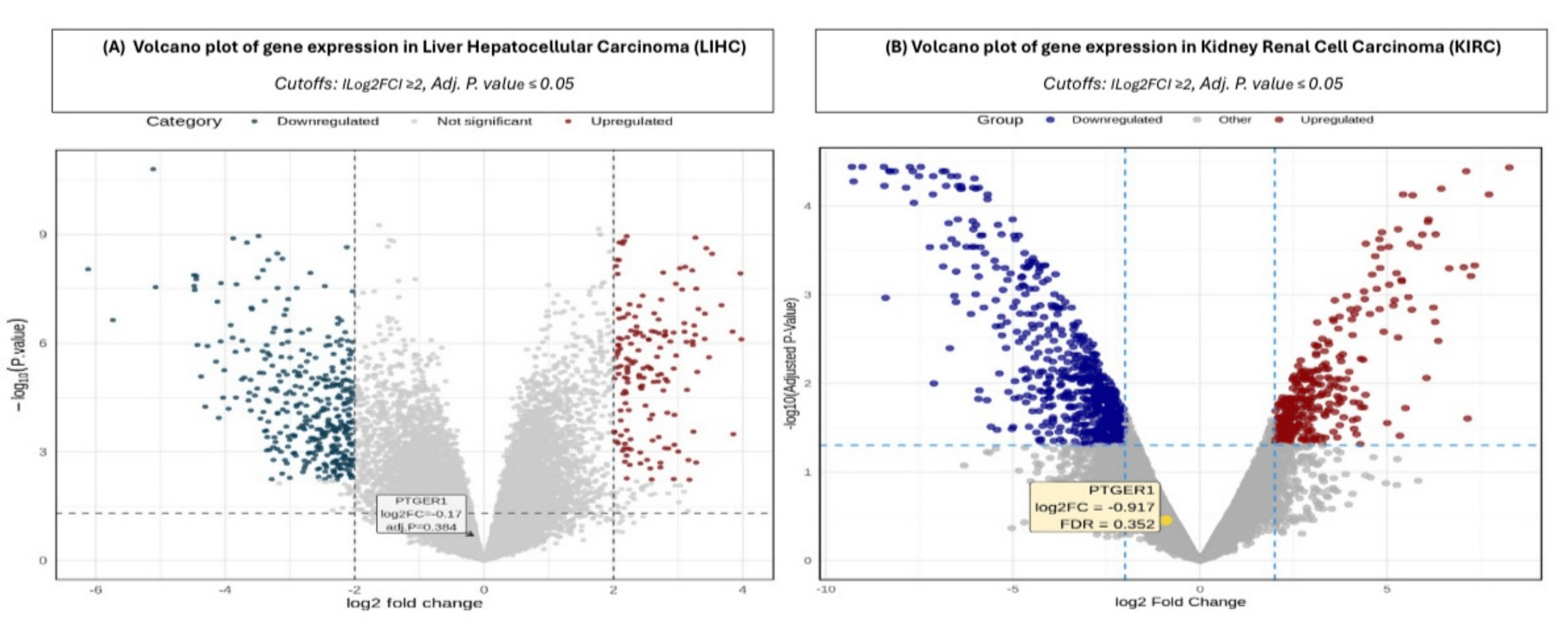
Volcano plots illustrating differential gene expression profiles in KIRC and LIHC with PTGER1 highlighted. (A) LIHC and (B) KIRC. Red: upregulated genes, Blue: downregulated genes KIRC: kidney renal cell carcinoma, LIHC: liver hepatocellular carcinoma, PTGER1: prostaglandin E receptor 1

To validate our previous findings across multiple cancer types, including KIRC, KICH, LIHC, and UCEC, we used the GEO database, where we conducted a targeted search focusing on human samples (*Homo sapiens*) and refined parameters focusing on expression profiling data derived from both microarray and mRNA sequencing technologies, ensuring reliable validation of our initial analysis. We assessed PTGER1 expression across two cancers: KIRC and LIHC, under a strict threshold of ILog2FCI>=2 and adjusted P-value < 0.05. Volcano plots demonstrating the relationship between fold change and adjusted P value were created using the R statistical computing program (R Foundation for Statistical Computing, Vienna, Austria), enabling visualization of DEGs across the studied cancer types. KIRC was analyzed via the GSE100666 dataset, comprising three tumor samples and three matched normal controls revealed 31,088 DEGs. Among these, 21,978 genes were downregulated (Log2FC < -2), and 9,110 were upregulated (Log2FC > 2). PTGER1 demonstrated non-significant downregulation with a Log2FC of -0.91678 and adjusted P-value of 0.352289. In LIHC, analysis of the GSE115018 dataset, which included 12 tumor samples and 12 para-cancerous normal tissue specimens collected 2 cm from the tumor margin, identified 26,684 DEGs. Of these, 192 genes were upregulated while 26,492 were downregulated. PTGER1 exhibited non-significant downregulation with a Log2FC of -0.16769602, adjusted P-value of 3.84e-01 (Figure 11). Validation using the GEO database was not performed for UCEC and KICH due to a lack of available datasets.

## Discussion

The PTGER family comprises four distinct prostaglandin E receptor subtypes (PTGER1-4), all of which belong to the G-protein coupled receptor superfamily and participate in diverse biological functions [2]. Our findings elucidate the role of EP2 in tumor immunology, persistent inflammatory response, neovascularization, and metastatic spread. Given its pro-inflammatory properties, pharmacological inhibition of EP2 represents a potential therapeutic approach for inflammation-driven pathology [9].

TCGA analysis via publicly available databases, GEPIA, UALCAN, and TIMER, demonstrated that PTGER1 was consistently downregulated in tumor samples when compared with normal samples in KICH and KIRC across the three databases. This supports its potential as a diagnostic biomarker in kidney tumors, while upregulation in tumor samples in UCEC and LIHC was observed in TIMER and UALCAN. Recent studies have highlighted the role of various prostanoid receptors in modulating inflammatory pathways, suggesting that dysregulation of PTGER1 could contribute to the tumor microenvironment's inflammatory state, potentially exacerbating cancer progression [10]. Further studies have shown that PTGER1 expression significantly affects renal function, particularly in the context of obstructed ureters, where its expression is normally elevated; downregulation may lead to a decrease in the inflammatory response mediated by prostaglandins, thereby influencing renal hemodynamics and the overall contractility of the ureters. Furthermore, elevated PTGER1 levels in obstructed ureters and stable expression across age groups were noted, suggesting a role in maintaining renal homeostasis and potential therapeutic relevance for urolithiasis and other renal pathologies. The interplay between prostaglandin receptors and inflammatory mediators likely influences tumor behaviour and treatment response, underscoring the importance of age-specific therapeutic strategies that account for distinct physiological and inflammatory profiles [11]. The downregulation of PTGER1 in malignancies raises questions about the interplay between prostaglandin signalling and cancer progression. Growing evidence suggests that PTGER3, another subtype of prostaglandin E receptors, exhibits differential expression in various cancers, such as cervical squamous cell carcinoma and endocervical adenocarcinoma (CESC), where its lower expression correlates with poor prognosis and recurrence-free survival [12].

UALCAN was used to investigate PTGER1 expression according to clinicopathological variables, including age, which is subclassified into young adults, middle-aged, older adults, and elderly, and by racial groups of Caucasian patients, African American patients, and Asian patients, and tumor stages in comparison to normal in KIRC, KICH, UCEC, and LIHC. PTGER1 is strongly expressed in normal kidney and consistently downregulated across all tumour stages in KICH and KIRC, supporting a tumor suppressive role. Minor stage-specific increase in KIRC (stages 2-4 vs. 2) does not alter the overall pattern. While a stage-dependent expression with distinct patterns was observed in UCEC, expression of PTGER1 increased progressively across stages, supporting a potential utility for late-stage detection. LIHC revealed transient upregulation in early/intermediate stage (stages 1 and 3) with a sharp decline in advanced disease, suggesting diagnostic value for early-stage liver cancer but limited applicability in advanced LIHC. Racial and ethnic background was evaluated in KIRC and KICH, showing consistent upregulation in normal tissue when compared with tumor samples in Caucasian patients, African American patients, and Asian patients. Conversely, PTGER1 expression in LIHC varies by race, being highest and most variable in African American patients. Furthermore, in UCEC, a race-dependent expression of PTGER1 was exhibited, being downregulated in Caucasian patients when compared with normal, upregulated in African American patients when compared with Caucasian and normal population, and the highest upregulation trend was observed in Asian patients. This is supported by previous reports, which indicated that gene expression levels of PTGER may vary across different racial and ethnic groups, potentially influencing how these populations respond to diseases and treatments [12]. Race-dependent PTGER1 expression may reflect broader ethnic differences in tumor biology, as African American patients with clear cell renal cell carcinoma (ccRCC) show distinct molecular profiles of lower VHL mutations, enriched ccB subtype associated with poorer outcomes compared to Caucasian patients [13]. Similarly, PTGER1 expression in KIRC and KICH was higher in normal tissues than in tumor samples when grouped by patient age. This downregulation in tumors may reflect a loss of PTGER1-mediated protective or anti-inflammatory signalling during malignant transformation. Conversely, in UCECE, expression of PTGER1 markedly increased with age, peaking in elderly patients, supporting its potential as an age-informed biomarker. Interestingly, PTGER1 expression was markedly higher in LIHC tissues from both middle-aged (41-60 years) and elderly (61-80 years) patients compared with normal controls, indicating that PTGER1 upregulation may be associated with age-related progression or susceptibility to hepatocellular carcinoma.

Methylation status was analyzed using the UALCLAN database in six cancers: KIRC, KICH, BLCA, LUAD, HNSC, and UCEC. In BLCA, normal tissues exhibited hypermethylation while tumor tissues displayed hypomethylation, coinciding with PTGER1 upregulation in malignant cells. Promoter methylation evolves with stages in KICH: early tumors show striking heterogeneity, while advanced tumors, especially stage IV, shift toward uniform hypermethylation. This trend points to a mechanism where epigenetic silencing enforces PTGER1 downregulation via CpG island DNA methylation, contrasting with its relatively higher expression in normal tissues. Furthermore, the dynamic nature of DNA methylation supports its utility as a biomarker for early cancer detection and prognosis, as aberrant methylation patterns can be detected in bodily fluids prior to clinical symptoms onset [14]. By contrast, in KIRC and LUAD, PTGER1 is hypermethylated in normal tissues, where it is also upregulated. In contrast, in HNSC, PTGER1 shows both hypermethylation and upregulation in tumor tissues. This pattern suggests that PTGER1 upregulation is not primarily driven by DNA methylation but may be regulated through alternative epigenetic mechanisms, such as histone modification [15]. Finally, in UCEC, tumors show promoter hypermethylation, indicating that PTGER1 upregulation in normal tissues is likely regulated by methylation. While hypermethylation is well known for silencing tumor suppressors, it also contributes to genomic instability, fuelling tumorigenesis, inactivating key DNA repair genes, such as hMLH1 and MGMT, GSTP1, and BRCA1, and accelerates mutation accumulation and cancer progression [16]. Interestingly, the methylation status of PTGER1 is not uniform across cancers, highlighting the dynamic ways in which epigenetic landscapes redirect gene expression during disease evolution [17]. Defining the epigenetic mechanism of cancer development enables targeted therapies to reverse genetic modifications and restore normal gene function [18]. Immune cell infiltration correlation with PTGER1 expression was analyzed in KIRC and KICH using the TIMER database to evaluate infiltration levels of CD8+ cytotoxic T cells, CD4+ helper T cells, neutrophils, macrophages, and dendritic cells. In KIRC, a significant weak positive correlation was noted with CD4+ T cells. Despite downregulation of PTGER1 expression, a weak immunological response may still drive tumor pathology. Subsequently, KIRC showed an insignificant weak positive correlation with CD4+, CD8+ T cells, and DC. In UCEC, PTGER1 expression showed a significant weak negative correlation with neutrophils and dendritic cells and an insignificant weak positive correlation with CD4⁺ T cells. Although associations with CD8⁺ T cells and macrophages were weak, negative, and insignificant, the overall downregulation expression pattern indicates a minor immunosuppressive influence of PTGER1 in the uterine tumor microenvironment via neutrophils and dendritic cells, weak inverse relationship with immune cells. In contrast, in LIHC, PTGER1 exhibited a significantly weak positive correlation with macrophages, implying involvement in macrophage recruitment or activation. The dynamics between T lymphocyte populations and tumor progression suggest that the immune microenvironment plays a critical role in influencing cancer outcomes. For instance, elevated levels of CD8+ T cells have been associated with improved disease-free survival in various cancers, including colorectal cancer, where a high presence of both CD4+ and CD8+ T cells correlates with better overall prognosis (p<0.05). Differentiation of CD4+ T cells into cytotoxic Th1 effector cells proved sufficient for mediating regression of established malignancies in major histocompatibility complex class II (MHC II)-dependent recognition [19]. Cancer proliferation, survival, and migration are dependent on inflammation, highlighting the immune microenvironment as a drive for tumor development and progression. Natural killer cells (NK) and dendritic cells are recognized to kill cancer cells directly via adaptive or innate immunity by antigen-presenting cells (APC); however, the presence of a tumor immune inhibitory environment contributes to impaired function and poor patient outcome [20]. CD8⁺ T cells exert cytotoxic effects against target cells through the release of interferon-γ (IFN-γ), interferon-α (IFN-α), and granzymes [21]. This is achieved by upregulating checkpoints like programmed cell death-1/programmed death ligand-1 (PD-1/PDL1) [22]. Tumor-associated neutrophils (TANs) are subdivided into anti-tumor (N1 TANs), by kill tumor cells via myeloperoxidase or histones, or pro-tumor (N2 TANs), enhancing proliferation through protease [23]. Beyond driving tumor expansion, PGE2 fundamentally alters the tumor microenvironment, establishing conditions that suppress anti-tumor immunity. Literature establishes that PGE2 impairs functional activation of immune effectors such as T lymphocytes and NK cells, enabling malignancies to escape immunological detection and elimination [24]. EP1, encoded by PTGER1, exhibits the lowest binding affinity for PGE2 among the prostaglandin E receptor subtypes. It triggers calcium channel entry into the cell via Gq protein coupling, while EP3 suppresses cyclic adenosine monophosphate (cAMP) through Gi inhibition of adenylate cyclase. In contrast, EP2 and EP4 elevate cAMP concentration by stimulating adenylyl cyclase through stimulatory G protein (Gs) signalling. The EP2/EP4 receptor axis functions as a master switch for PGE2-driven inflammation and tumorigenesis [25]. The ability of PGE2 to suppress immune function provides a strong justification for therapeutic intervention that disrupts this signalling axis to maximize immune system engagement against tumors [26]. This raises the question whether reduced PTGER1 expression in KIRC and KICH could indicate a context-dependent role of EP1, potentially reflecting a shift away from immune modulation toward alternative tumor-promoting pathways. Consequently, these data imply that immune suppression might not be the primary driver of tumor progression in kidney cancers where PTGER1 is diminished.

Survival analysis identified PTGER1 as a clinically relevant prognostic indicator with cancer type specificity. KIRC and LUSC demonstrate the most compelling evidence for EP1 expression correlating with adverse clinical outcome, validated through triple database concordance: GEPIA, UALCAN, and Kmplot. Additional malignancies, including UCEC, LGG, UVM, and KIRP, warrant prospective investigation given preliminary two-platform support. The protective trend observed in LIHC challenges uniform interpretation and points to organ-specific prostaglandin signalling dynamics. Overall, these results position PTGER1 as a candidate stratification tool requiring cancer-selective application and expanded clinical validation.

In CbioPortal, PTGER1 genomic profiling across the TCGA database revealed an overall alteration frequency of approximately 2-3% with gene amplification predominating over deletion and mutation, indicating EP1 receptor overexpression through DNA replication producing amplicons rather than loss of function drives its oncogenic role [27,28]. We observed that gynaecological malignancies exhibit the highest alteration rate, particularly endometrial and ovarian cancer, consistent with established prostaglandin hormone signalling interaction in these tissues. This is reinforced by studies demonstrating that PTGER2 in ovarian cancer is identified as an oncogene that promotes cancer stem cell properties and resistance to chemotherapy, resulting in poor prognosis [29]. Other evidence may strengthen our findings as PTGER2 and PTGER4 were found to play a key role in endometriosis development, and their inhibition leads to poor prognosis [30]. Notably, hepatocellular carcinoma displays a uniquely heterogeneous alteration profile encompassing amplification, deletion, structural variants, and mutations, which may explain the paradoxical impact on disease progression; however, this warrants further research.

Limitations

This study provides an overview of PTGER1 expression across multiple cancers; however, it is purely an in-silico design and does not include experimental evidence to support the computational results based on publicly available datasets and bioinformatics platforms, including UALCAN, GEPIA, and TIMER, which may introduce analytical variations and potential biases. Heterogeneity of platforms, batch effects, and inconsistent sample sizes across datasets could also influence the results. In addition, weak correlations observed in some analyses may be prone to overinterpretation. Laboratory-based studies, including gene manipulation and functional assays in cell or animal models, are needed to verify whether PTGER1 directly influences tumor behaviour or immune regulation.

## Conclusions

PTGER1, a prostaglandin E2 receptor, shows promise as a diagnostic biomarker in KIRC and KICH and a poor prognostic biomarker in KIRC and LUSC, with robust validation across three publicly available databases: UALCAN, TIMER, and GEPIA, correlating with immune infiltration profiles, indicating its potential utility in risk stratification and patient management. Additionally, PTGER1 is associated with altered immune cell infiltration patterns, suggesting that PTGER1 may play a role in modulating the tumor immune microenvironment and can serve as a target for immune therapeutic interventions. PTGER1 also demonstrates potential as a poor prognostic biomarker in UCEC, UVM, LGG, and KIRP. Interestingly, it appears to function as a favourable prognostic biomarker in LIHC. However, these findings were confirmed by only two databases, which limit their reliability and necessitate further validation through additional independent cohorts and experimental studies before clinical implementation.
